# Evidence of Compromised Blood-Spinal Cord Barrier in Early and Late Symptomatic SOD1 Mice Modeling ALS

**DOI:** 10.1371/journal.pone.0001205

**Published:** 2007-11-21

**Authors:** Svitlana Garbuzova-Davis, Samuel Saporta, Edward Haller, Irina Kolomey, Steven P. Bennett, Huntington Potter, Paul R. Sanberg

**Affiliations:** 1 Center of Excellence for Aging & Brain Repair, College of Medicine, University of South Florida, Tampa, Florida, United States of America; 2 Department of Neurosurgery, College of Medicine, University of South Florida, Tampa, Florida, United States of America; 3 Department of Molecular Pharmacology and Physiology, College of Medicine, University of South Florida, Tampa, Florida, United States of America; 4 Department of Pathology and Cell Biology, College of Medicine, University of South Florida, Tampa, Florida, United States of America; 5 Department of Psychiatry, University of South Florida, College of Medicine, Tampa, Florida, United States of America; 6 Department of Molecular Medicine, College of Medicine, University of South Florida, Tampa, Florida, United States of America; 7 Johnnie B. Byrd's Alzheimer Center and Research Institute, Tampa, Florida, United States of America; Uppsala University, Sweden

## Abstract

**Background:**

The blood-brain barrier (BBB), blood-spinal cord barrier (BSCB), and blood-cerebrospinal fluid barrier (BCSFB) control cerebral/spinal cord homeostasis by selective transport of molecules and cells from the systemic compartment. In the spinal cord and brain of both ALS patients and animal models, infiltration of T-cell lymphocytes, monocyte-derived macrophages and dendritic cells, and IgG deposits have been observed that may have a critical role in motor neuron damage. Additionally, increased levels of albumin and IgG have been found in the cerebrospinal fluid in ALS patients. These findings suggest altered barrier permeability in ALS. Recently, we showed disruption of the BBB and BSCB in areas of motor neuron degeneration in the brain and spinal cord in G93A SOD1 mice modeling ALS at both early and late stages of disease using electron microscopy. Examination of capillary ultrastructure revealed endothelial cell degeneration, which, along with astrocyte alteration, compromised the BBB and BSCB. However, the effect of these alterations upon barrier function in ALS is still unclear. The aim of this study was to determine the functional competence of the BSCB in G93A mice at different stages of disease.

**Methodology/Principal Findings:**

Evans Blue (EB) dye was intravenously injected into ALS mice at early or late stage disease. Vascular leakage and the condition of basement membranes, endothelial cells, and astrocytes were investigated in cervical and lumbar spinal cords using immunohistochemistry. Results showed EB leakage in spinal cord microvessels from all G93A mice, indicating dysfunction in endothelia and basement membranes and confirming our previous ultrastructural findings on BSCB disruption. Additionally, downregulation of Glut-1 and CD146 expressions in the endothelial cells of the BSCB were found which may relate to vascular leakage.

**Conclusions/Significance:**

Results suggest that the BSCB is compromised in areas of motor neuron degeneration in ALS mice at both early and late stages of the disease.

## Introduction

The central nervous system (CNS) is an immunologically privileged zone, which is normally protected from entry of immune cells and serum proteins by the blood-brain barrier (BBB), blood-spinal cord barrier (BSCB), and blood-cerebrospinal fluid barrier (BCSFB). These barriers are specialized structures of the CNS that control cerebral/spinal cord homeostasis by selective transport of molecules and cells from the systemic compartment (reviewed in [1]–[7]). This control is possible due to the unique structural elements of the microvasculature – endothelial cells of brain capillaries and epithelial cells of the choroids plexus, astrocyte end-feet, and pericytes. Brain (spinal cord) capillary endothelial cells are distributed along the length of the vessels, completely encircling the lumen and connected via adherens and tight junctions. Adherens junctions support intercellular adhesion, as tight junctions form a diffusion barrier for most blood-borne substances. The basement membrane (i.e. basal lamina), surrounding the endothelial cells and pericytes, supports the abluminal surface of the endothelium. The basement membrane, which consists of laminin, fibronectin, collagens, and proteoglycans [8], [9], separates adjacent tissues, acting as a barrier to the passage of macromolecules and cell migration. The astrocyte perivascular end-feet ensheathing approximately 95% of the vessel wall appear to have an important role for maintenance of the BBB [10]. Thus, functional integrity of all BBB/BSCB elements is critical for protection of the CNS from various harmful blood substances.

Impairment of the BBB occurs in various pathological CNS conditions. Accumulation of collagen in vascular basement membranes and focal necrotic changes in endothelial cells were found in Alzheimer's patients [11]. Degradation of the extracellular matrix may be concomitant with BBB disruption and tissue softening, setting the stage for the most pronounced forms of brain swelling and leading to the development of severe cerebral edema over subsequent hours and days in stroke patients (reviewed in [12]). In multiple sclerosis (MS), which is characterized by inflammatory lesions within the CNS, BBB disruption enables leakage of the serum protein fibrinogen into the brain parenchyma [13], [14] and may precede myelin damage aggravating the inflammatory process. Recently, Vos et al. [15] demonstrated that BBB dysfunction in MS patients is apparent not only in focal lesions but also in diffuse abnormalities in white matter which were detected by postmortem MRI. BSCB breakdown was found in an experimental model of traumatic spinal cord injury [16], [17]. It has been shown that spinal nerve lesions also alter BSCB function [18]. Results of these studies showed increased microvascular permeability leading to blood protein extravasation and the formation of vasogenic edema that play important roles in the pathophysiology of the diseased or injured spinal cord.

Amyotrophic lateral sclerosis (ALS) is a progressive degenerative disease affecting motor neurons in the spinal cord, motor cortex and brainstem that leads to paralysis and death within five years of disease onset [19], [20]. Although numerous hypotheses about the etiology and pathogenesis of ALS have been proposed (reviewed in [21]–[23]), increasing evidence points to immune system involvement in ALS pathogenesis [24]–[27]. In the spinal cord and brain of both ALS patients and animal models, the presence of T-cell lymphocytes [25], [28], deposits of IgG [29]–[32], complement components C3 and C4 [29], and monocyte/macrophage and dendritic cells [28], [33], [34] were observed that may have a critical role in motor neuron damage. Recently, IgG was detected in the perikarya of motor neurons of the lumbar spinal cord in mice after intraperitoneal injection of IgG derived from sera of ALS patients [31]. Moreover, the uptake of IgG in multivesicular bodies in endothelial cells in the affected areas of the spinal cord was found in both ALS patients and mice injected with human ALS IgG.

Additionally, significantly increased levels of albumin, IgG, and C3c have been noted in the cerebrospinal fluid of ALS patients [35]–[38]. These findings suggest that barrier permeability may be affected in ALS. Recently, we showed disruption of the BBB and BSCB in areas of motor neuron degeneration in the brain and spinal cord in G93A SOD1 mice modeling ALS at both early and late stages of disease using electron microscopy [39]. Examination of capillary ultrastructure revealed endothelial cell degeneration, which, along with astrocyte alteration, compromised the BBB and BSCB. However, the effect of these alterations upon barrier function in ALS is still unclear. The aim of this study was to determine the functional competence of the BSCB in G93A SOD1 mice modeling ALS at different stages of disease.

## Results

### Disease symptom progression in G93A mice

**Figure 1:**
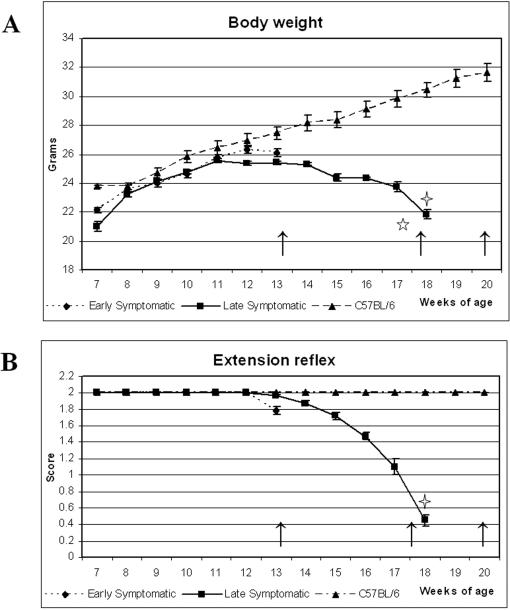
Characteristics of disease progression in G93A mice. (A) Body weight and (B) extension reflex of G93A and control C57BL/6J mice. G93A mice at about 13 weeks of age showed initial signs of disease such as weight loss and reduced hindlimb extension. Terminal stage of disease was observed at 17–18 weeks of age, as demonstrated by complete hindlimb paralysis, significant reduction of body weight and absence of hindlimb extension. Arrows indicate the age of mice when euthanatasia was performed. The five pointed star in A indicates difference (p = 0.06) in body weights between G93A mice at 13 weeks of age and 18 weeks of age; the four pointed star indicates a significant difference (p = 0.007) in body weights between G93A and C57BL/6J mice at 18 weeks of age. The four pointed star in B indicates a significant difference in extension reflex (p<0.001) between G93A mice at 13 weeks of age and 18 weeks of age.

The G93A mice were monitored weekly for initial disease symptoms and disease symptom progression by body weight and extension reflex, beginning at 7 weeks of age. Body weights of G93A mice gradually increased until 11 weeks of age and then stabilized for 2 weeks (12–13 weeks of age). Tremor, an initial sign of disease, was seen in some mice as early as 13 weeks of age. After this time, mice consistently lost body weight due to muscle atrophy. By 18 weeks of age, G93A mice had lost approximately 14% (p = 0.06) of their 13-weeks-of-age body mass, dropping from 25.4±0.38 g to 21.8±0.55 g (Figure 1, A). Eighteen weeks old G93A mice weighed 28% less than C57BL/6J mice of the same age (p = 0.007), which continued to maintain their body weights until 20 weeks of age. Deterioration in extension reflex started to appear in G93A mice at 13 weeks of age (Figure 1, B). During the next 5 weeks, mice demonstrated slowly declining hindlimb extension and by 18 weeks of age, showed no extension (p<0.001) and exhibited hindlimb paralysis. Thus, G93A mice at about 13 weeks of age showed initial signs of disease such as tremor, weight loss, and reduced hindlimb extension. Terminal stage of disease in these mice was observed at 17–18 weeks of age, as demonstrated by complete hindlimb paralysis, significant reduction of body weight and absence of hindlimb extension.

### Motor neurons in the cervical and lumbar spinal cords of G93A mice

**Figure 2:**
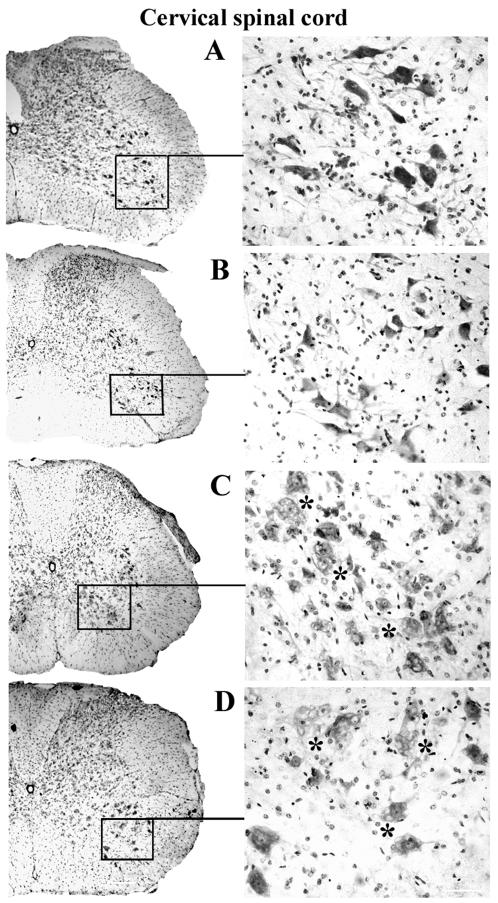
Motor neurons in the cervical spinal cord of G93A mice at early and late stage of disease (cresyl violet staining). In the cervical spinal cord, many healthy motor neurons with large soma and neuritic processes were identified in the control C57BL/6J mice at (A) 12–13 weeks of age and (B) 19–20 weeks of age. In G93A mice, numerous motor neurons with vacuolization (asterisks) were found at (C) 13 weeks of age and (D) decreased numbers of motor neurons were noted in 17–18 week old mice. Motor neurons of various sizes displayed vacuolization (asterisks). Scale bar on left side is 200 µm, right side is 50 µm.

**Figure 3:**
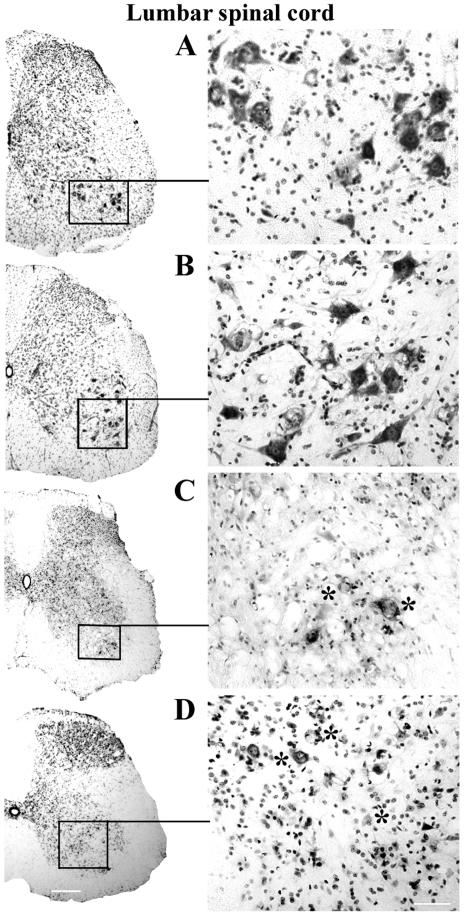
Motor neurons in the lumbar spinal cord of G93A mice at early and late stage of disease (cresyl violet staining). In the lumbar spinal cord, C57BL/6J mice at (A) 12–13 weeks of age and (B) 19–20 weeks of age showed numerous motor neurons with strong Nissl body staining. Most degenerated or swollen motor neurons (asterisks) were found in G93A mice at (C) early (13 weeks of age) and (D) late (17–18 weeks of age) stages of disease; most surviving motor neurons were small. Scale bar on left side is 200 µm, right side is 50 µm.

Nissl body staining was performed to identify motor neuron condition in the spinal cords of G93A mice in early and late stages of disease. In control C57BL/6J mice, many healthy motor neurons with large soma and neuritic processes were visible in the cervical and lumbar ventral horn of the spinal cords at 12–13 weeks of age (Figure 2A, Figure 3A) and 19–20 weeks of age (Figure 2B, Figure 3B). In the cervical spinal cord of G93A mice, numerous motor neurons with vacuolization were found at 13 weeks of age, concurrent with initial disease symptoms (Figure 2C). When disease symptoms progressed and mice were paralyzed, usually at 17–18 weeks of age, the number of motor neurons had decreased and motor neurons of various sizes displayed vacuolization (Figure 2D). Only a few healthy motor neurons were identified at this time. More dramatic changes in motor neuron condition were found in the lumbar spinal cords of ALS mice. Most motor neurons in 13 week old mice showed signs of degeneration (Figure 3C). In G93A mice at 17–18 weeks of age, the ventral horn was essentially devoid of motor neurons (Figure 3D).

### Fluorescent detection of Evans Blue in the cervical and lumbar spinal cords of G93A mice

**Figure 4:**
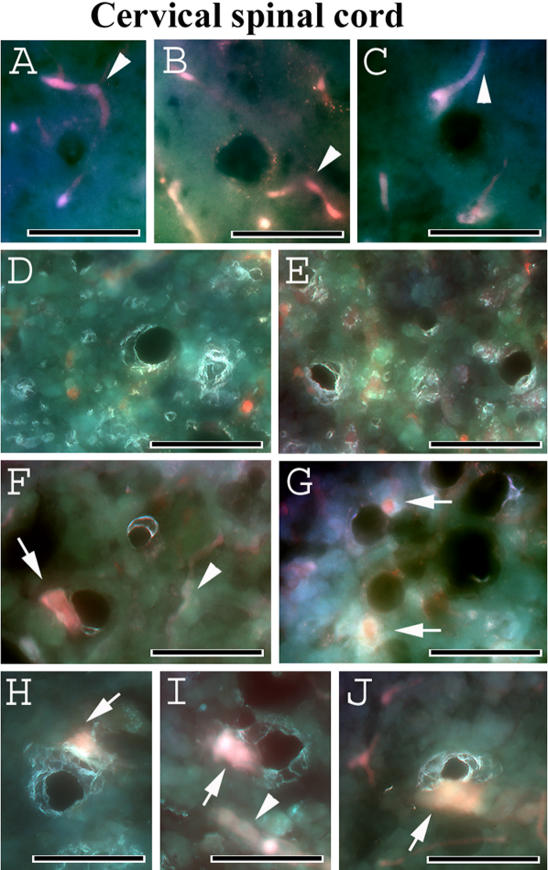
Evans Blue fluorescence in the cervical spinal cord of G93A mice at early and late stages of disease. In the cervical spinal cord, EB was clearly detected within the blood vessels (red, arrowheads) in the control C57BL/6J mice at (A, B, C) 12–13 weeks of age or (D, E) in the lumen of vessels (brilliant green) at 19–20 weeks of age. In G93A mice, vascular leakage of EB (red, arrows) was detected (F, G) at early (13 weeks of age) disease symptoms and (H, I, J) at end-stage of disease (17–18 weeks of age) when more EB extravasation was seen. Arrowheads in F and I indicate vessel permeability. Scale bar in A–J is 25 µm.

**Figure 5:**
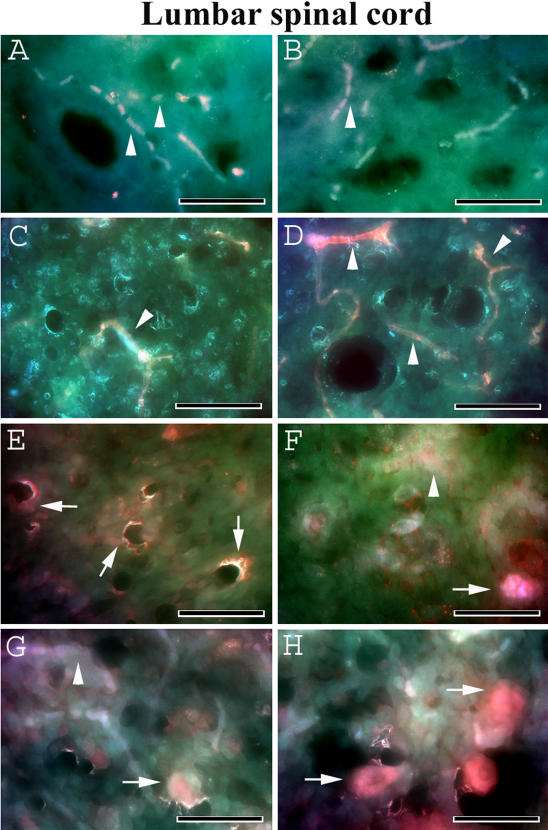
Evans Blue fluorescence in the lumbar spinal cord of G93A mice at early and late stages of disease. In the lumbar spinal cord, EB dye (red, arrowheads) was determined intravascularly in the control C57BL/6J at (A, B) 12–13 weeks of age and (C, D) 19–20 weeks of age similar to the cervical spinal cord. EB extravasation abnormalities were found in G93A mice at (E, F) 13 weeks of age (red, arrows). (G, H) Significant EB diffusion (red, arrows) into the parenchyma of the lumbar spinal cord from many blood vessels was detected in G93A mice at end-stage of disease (17–18 weeks of age). Arrowheads in F and G indicate vessel permeability. Scale bar in A–H is 25 µm.

Evans Blue, the tracer used to assess disruption of the BSCB, was injected into G93A mice at initial (13 weeks of age) and late (17–18 weeks of age) stages of disease symptoms prior to euthanasia. C57BL/6J mice were also administered the EB dye. In the cervical spinal cord, EB was clearly detected within the blood vessels in the control C57BL/6J mice at 12–13 weeks of age (Figure 4A, B, C) or in the cross-sectioned capillaries at 19–20 weeks of age (Figure 4D, E). Vascular leakage of EB was distinguished in G93A mice with early disease symptoms (Figure 4F, G) and at end-stage of disease (Figure 4H, I, J) when more EB extravasation was noted. Interestingly, vessel permeability was perceived in both initial (Figure 4F) and late (Figure 4I) symptomatic G93A mice. In the lumbar spinal cord, EB dye was observed intravascularly in control C57BL/6J mice at 12–13 weeks of age (Figure 5A, B) and 19–20 weeks of age (Figure 5C, D), similar to results in the cervical spinal cord, while EB extravasation abnormalities were found in G93A mice at 13 weeks of age (Figure 5E, F). Significant EB diffusion into the parenchyma of the lumbar spinal cord from many blood vessels was detected in G93A mice at end-stage of disease, 17–18 weeks of age (Figure 5G, H). Considerable vessel permeability was seen in early (Figure 5F) as well as late (Figure 5G) symptomatic G93A mice. Thus, vascular leakage of EB was detected in G93A mice not only at end-stage of disease but also concurrent with early disease symptoms.

### Immunochistochemical characteristics of basement membranes, endothelial cells, and astrocytes in the spinal cords of G93A mice

**Figure 6:**
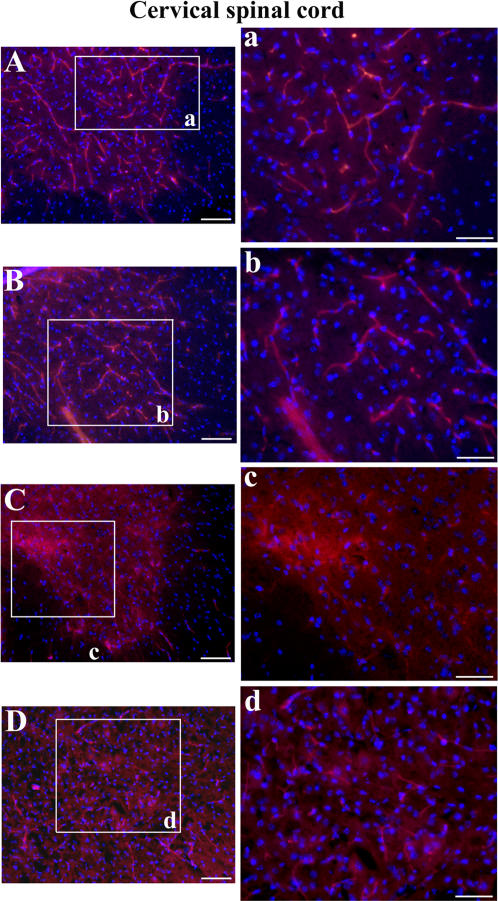
Immunofluorescence staining for laminin in the cervical spinal cord of G93A mice at early and late stages of disease. Many blood vessels of different diameter were immunoreactive for laminin-1 (red) in the control C57BL/6J mice at (A) 12–13 weeks of age and (B) 19–20 weeks of age. In G93A mice at (C) initial or (D) late stages of disease, capillaries appear to be less numerous. In some early symptomatic G93A mice, (C) blurry spots around capillaries were found. The nuclei in A–D are shown with DAPI. Scale bar in A, B, C, D is 200 µm; inserts a, b, c, d is 50 µm.

**Figure 7:**
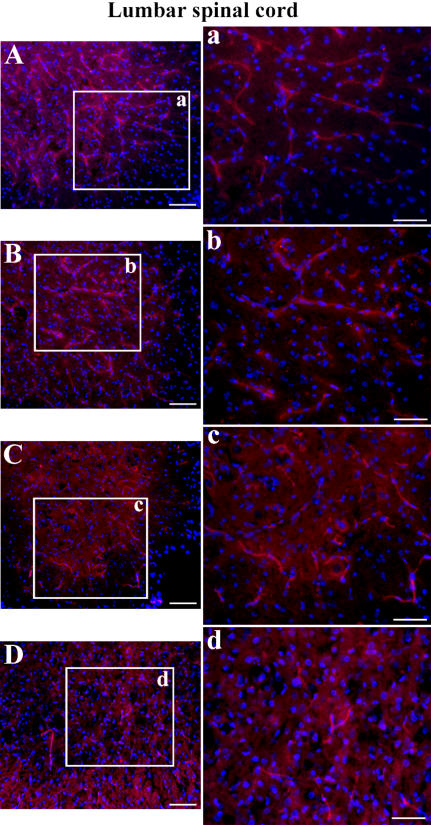
Immunofluorescence staining for laminin in the lumbar spinal cord of G93A mice at early and late stages of disease. Various laminin-positive vessels (red) were observed in the control C57BL/6J mice at (A) 12–13 weeks of age and (B) 19–20 weeks of age similar to cervical spinal cord results. Fewer blood vessels were labeled in G93A mice at (C) early or (D) end-stage of disease. The nuclei in A–D are shown with DAPI. Scale bar in A, B, C, D is 200 µm; inserts a, b, c, d is 50 µm.

Immunofluorescent staining for laminin-1 (major non-collagenous basement membrane glycoprotein) demonstrated well organized microvasculature networks in the cervical and lumbar ventral horn of the spinal cords in C57BL/6J mice at 12–13 weeks of age (Figure 6A, Figure 7A) and 19–20 weeks of age (Figure 6B, Figure 7B). In these control animals, many capillaries of different calibers were visible by laminin detection. Conversely, a marked reduction of labeled vessels was observed in both cervical and lumbar spinal cords of G93A mice at early (Figure 6C, Figure 7C) and late (Figure 6D, Figure 7D) stages of disease, suggesting a loss of vascularization or disruption of vascular basement membrane integrity. In some cervical spinal cord sections of G93A mice with initial disease symptoms, blurred spots around capillaries were apparent in the ventral horn of the spinal cord (Figure 6C), probably due to vascular leakage.

**Figure 8:**
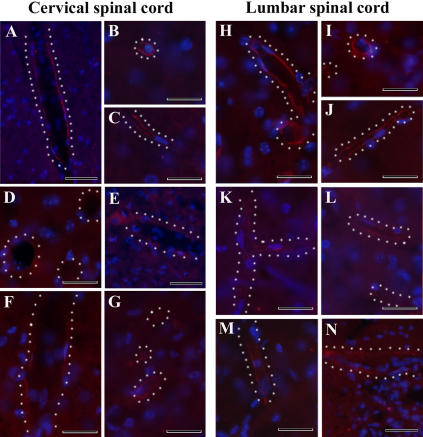
Immunofluorescence staining for Glut-1 in the cervical and lumbar spinal cords of G93A mice at early and late stages of disease. *Cervical spinal cord.* High expression of Glut-1 (red) was determined in endothelial lining of many blood vessels of various diameters in the cervical spinal cord of the control C57BL/6J mice at (A), (B) 12–13 weeks of age and (C) 19–20 weeks of age. In G93A mice at (D), (E) initial or (F), (G) late stages of disease, immunoreaction for Glut-1 in the endothelial cells appear to be low, or nonexistent. The nuclei in A–G are shown with DAPI. Scale bar in A and E is 50 µm; B, C, D, F, G is 25 µm. Outline of white dots indicates configuration of blood vessels. *Lumbar spinal cord.* Similar to the cervical spinal cord, most Glut-1-positive endothelial cells (red) were observed in the control C57BL/6J mice at (H), (I) 12–13 weeks of age and (J) 19–20 weeks of age. Less Glut-1 expression was found in G93A mice at (K), (L) early or (M), (N) end-stage of disease. The nuclei in H–N are shown with DAPI. Scale bar in J, M, N is 50 µm; H, I, K, L is 25 µm.

Immunofluorescent staining for Glut-1 (glucose transporter 1) showed high expression of Glut-1 in microvascular endothelia of the cervical and lumbar ventral horn of the spinal cords in C57BL/6J mice at 12–13 weeks of age (Figure 8A, B, H, I) and at 19–20 weeks of age (Figure 8C, J). The Glut-1 immunoreaction of endothelial cells lining the many capillaries of different calibers was low, or none, in both cervical and lumbar spinal cords of G93A mice at early (Figure 8D, E, K, L) and late (Figure 8F, G, M, N) stages of disease.

**Figure 9:**
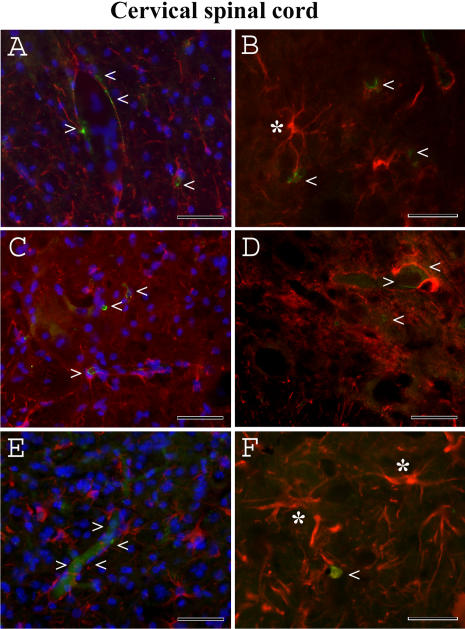
Immunohistochemical staining for endothelial cells (CD146) and astrocytes (GFAP) in the cervical spinal cord of G93A mice at early and late stages of disease. (A, B) Normal appearance of endothelial cells (green, arrowheads) and delineated astrocytes (red, asterisk) was observed in the control C57BL/6J mice at 19–20 weeks of age. Endothelia (green, arrowheads) surrounding capillaries were partially revealed in G93A mice at (C, D) initial or (E, F) late stages of disease. Note: increased astrocyte activation in the cervical spinal cord (F, asterisks) was detected in G93A mice at late stage of disease. The nuclei in A, C, and E are shown with DAPI. Scale bar in A, C, E is 50 µm; B, D, F is 25 µm.

**Figure 10:**
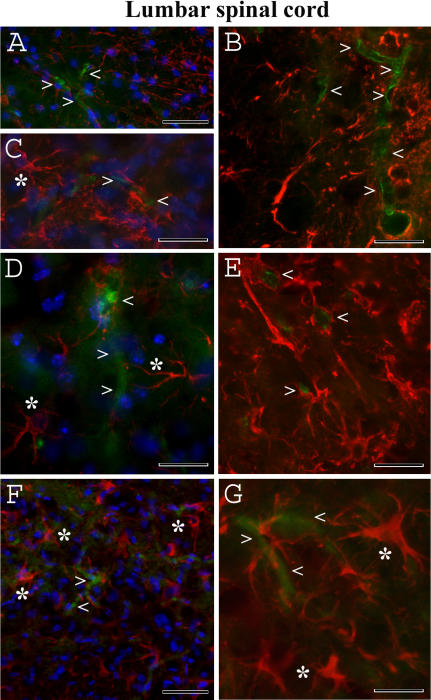
Immunohistochemical staining for endothelial cells (CD146) and astrocytes (GFAP) in the lumbar spinal cord of G93A mice at early and late stages of disease. (A, B, C) Similar to cervical spinal cord, endothelial cells (green, arrowheads) and astrocytes (red, asterisk) in C57BL/6J mice at 19–20 weeks of age appeared normal. In G93A mice at (D, E) early or (F, G) end-stage of disease, decreased CD146 antigen expression by endothelial cells (green, arrowheads) was observed. Note: increased astrocyte activation in the lumbar spinal cord (F, G, asterisks) was detected in G93A mice at late stage of disease. The nuclei in A, C, D, and F are shown with DAPI. Scale bar in A, C, D, F is 50 µm; B, E, G is 25 µm.

Immunohistochemically, endothelial cells (CD146) and astrocytes (GFAP) were of normal appearance in the cervical and lumbar ventral horn of the spinal cords in the control C57BL/6J mice at 12–13 weeks of age (data not shown) and 19–20 weeks of age (Figure 9A, B; Figure 10A, B, C). Delineated astrocytes and astrocytes with perivascular end-feet on the vessel wall were clearly observed. However, endothelia surrounding capillaries were partially revealed in the cervical spinal cord of G93A mice at initial (Figure 9C, D) or late (Figure 9E, F) stages of disease. Although CD146 antigen expression was detected in some endothelial cells of early symptomatic G93A mice, indistinct immunoreaction for CD146 was observed in G93A mice at end-stage of disease. Additionally, swollen endothelial cells (Figure 9F) were found in the cervical spinal cord of late symptomatic G93A mice. A small number of delineated astrocytes were also noted. In the lumbar spinal cord of G93A mice at initial (Figure 10D, E) and late (Figure 10F, G) stages of disease, decreased or unclear CD146 antigen expression was noted in endothelial cells. Notably, increased astrocyte activation in both cervical (Figure 9F) and lumbar (Figure 10F, G) spinal cords was detected in G93A mice at late stage of disease.

## Discussion

In the present study, we investigated the functional competence of the BSCB in G93A SOD1 mice modeling ALS at different stages of disease. We observed microscopic evidence of BSCB impairment in cervical and lumbar spinal cords, areas of motor neuron degeneration, of ALS mice at initial disease symptoms and, more severely, at late stage disease. Our data show EB leakage in cervical/lumbar spinal cord microvessels in G93A mice at early (13 weeks of age) and late (17–18 weeks of age) stage disease. More leakage was found in lumbar spinal cords of mice at terminal stage disease. Additionally, basement membrane disruption was noted at both early and late stage disease, as shown by the loss of laminin staining in the G93A mice. Downregulation of Glut-1 and CD146 expressions in spinal cord endothelial cells was also found in G93A mice at early and late stage disease and may relate to altered endothelial lining leading to vascular leakage. Small numbers of delineated astrocytes were also established. These results confirm our previous ultrastructural findings [39] on disruption of the BSCB showing functional incompetence of BSCB structural elements in ALS mice.

Significant death of motor neurons in G93A mice occurs at the onset of clinical disease (90 days) and by end-stage disease (136 days), mice show up to a 50% loss of cervical and lumbar motor neurons [40]–[42]. In G93A mice, motor deficits have been observed in tests of muscle strength and coordination as early as 8 weeks of age [43]. These results extend those of our previous studies [44]–[46], showing initial signs of disease, such as tremor, weight loss, and reduced hindlimb extension, in G93A mice at about 13 weeks (90 days) of age. At this age, numerous vacuolizated motor neurons were found in the cervical lumbar spinal cord and most motor neurons in the lumbar spinal cord showed signs of degeneration.

The primary BBB/BSCB function is control of the CNS homeostasis by selective transport of molecules and cells from the systemic compartment. Substances with a molecular weight higher than 400 Da generally cannot cross the barriers by free diffusion. However, certain endogenous large molecules, such as insulin, leptin, transferrin, and insulin-like growth factors, enter the brain from blood via specific endothelial carrier-mediated or receptor-mediated transporters (reviewed in [1], [2], [4], [47]). Recently, IgG was detected in the perikarya of motor neurons of the lumbar spinal cord in mice 24 hours after intraperitoneal injection of IgG derived from sera of ALS patients [31]. The injected IgG was found in the axon terminals of the lumbar ventral horn motor neurons, localizing in the microtubules and rough endoplasmic reticulum. Furthermore, IgG was similarly detected in spinal cord motor neurons of ALS patients. There was also evidence of IgG intake in endothelial cells in affected areas of the spinal cord in both ALS patients and mice injected with human ALS IgG. Engelhardt et al. [31] suggest that “there may be multiple antibodies targeting a variety of epitopes of motor neurons in ALS”. The molecular weight of IgG is 150,000 Da and it is unlikely that these molecules could cross an intact brain capillary endothelium even by receptor-mediated transcytosis. However, Pirttila et al. [48] showed that insulin-like growth factor (IGF)-1, IGF binding protein-2, or nitric oxide were not elevated in CSF of ALS patients, suggesting that there is not a major disruption in the BCSFB. In another report [49], an ALS mouse model with a permissive BBB was created by crossing G93A mouse with the *mdr1a/b* knockout mouse and showed that cyclosporine A (CsA), which cannot cross an intact BBB, BSCB or BCSFB, reached the CNS when delivered intraperitoneally into this combined mouse model. Since the authors did not investigate BBB or BSCB condition in the original transgenic G93A mice, it is possible that disruption or dysfunction of these barriers occur in ALS. The 1200 Da molecular weight of CsA is much smaller than that of IgG. Further investigation is needed to resolve this apparent discrepancy in ability to cross the BBB/BSCB.

Our finding of Evans blue extravasion in early symptomatic G93A mice may suggest that large molecules such as IgG and other blood proteins appear in the spinal cord due to vascular leakage, one possible mechanism accelerating motor neuron damage. However, it is unclear if BBB/BSCB disruption appears prior to motor neuron degeneration or as result of motor neuron dysfunction. Also, differences between the BBB and BSCB in endothelial protein concentrations may impact observed pathological changes in G93A mice. It has been shown that microvascular endothelial cells, isolated from murine spinal cord, morphologically similar to BBB endothelial cells, express reduced amounts of several prominent BBB proteins such as tight junction-associated proteins ZO-1 and occluding, adherens junction-associated proteins beta-catenin and VE-cadherin, and the efflux transporter P-glycoprotein [50].

Reduction in immunofluorescent labeling of basement membrane of affected G93A mice suggests possible membrane disruption. The basement membrane is part of the extracellular matrix and is composed of collagens, proteoglycans, elastin and several glycoproteins, of which laminin is the most abundant [8], [9]. Reduced laminin labeling was observed in cervical and lumbar spinal cords of both early and late symptomatic G93A mice, possibly indicating vascularization changes leading to capillary wall permeability. Interestingly, Ono et al. [51] showed fragmented and widely separated collagen bundles in capillaries and decreased amounts of collagen in postmortem posterior half of the lateral funiculus and in the anterior horn of cervical enlargements from patients with sporadic ALS. Although the role of these aberrations in the pathogenesis of ALS remains to be determined, the authors suggested that abnormalities of collagen in the perivascular spaces of capillaries “may be secondary to neuronal degeneration as a underlying mechanism in ALS”.

It is well known that glucose transport through the BBB (BSCB) is mediated by glucose transporter isoform 1 (Glut-1) [52], [53]. Glut-1 is associated mainly with the brain capillary endothelial cells and is asymmetrically distributed between the luminal and abluminal membranes [54]. This asymmetric intracellular pool of glucose transporter may provide for rapid transport of glucose across the abluminal plasmalemma to the brain parenchyma [55]. The alteration of Glut-1 may be related to the pathogenesis of microvascular permeability as has been shown, for example, in cerebral edema (reviewed in [56]). In the present study, we found low and mostly absent expression of Glut-1 in capillaries of both cervical and lumbar spinal cords of G93A mice at early and late stages of disease. This downregulation of Glut-1 expression in the endothelial cells of the BSCB may be related to altered endothelial lining leading to vascular leakage. Alternatively, decreased Glut-1 expression may result from aggravated alterations of the BSCB in G93A mice. Although additional experiments such as quantitative analysis of Glut-1 distribution and density in the endothelial plasma membranes are needed to elucidate the regulatory mechanisms of Glut-1 expression in the spinal cord, the present study indicates that alteration of Glut-1 could be involved in the pathogenesis of ALS.

Another of our findings was that endothelia surrounding capillaries were partially revealed by CD146 antigen expression in the cervical and lumbar spinal cords of G93A mice at initial and, more markedly, at late stages of disease. Moreover, small numbers of delineated astrocytes were established. These results may indicate that degeneration or, at least, partial dysfunction, of non-neuronal cells in ALS occurs. Evidence of widespread inflammatory reactions in ALS already exists. The presence of monocyte/macrophage cells, activated microglia, and reactive astrocytes was established in the spinal cord tissue of most ALS patients [28], [33], [34], [57], [58]. In a mouse model of ALS, immune/inflammatory responses [59], [60] are present even before any evidence of motor dysfunction [25], [61]. Strategically, astrocytes are located at the interface between the blood vessels and the brain as well as in the spinal cord parenchyma, influencing both the entry of blood cells into the CNS and the activity of invading cells once they have entered the brain parenchyma (reviewed in [62], [63]). It has been shown that activated astrocytes and microglia “in response to signals derived from the immune system or generated within the CNS” produce various inflammatory molecules that may increase the permeability of the endothelial cell barrier [10]. Inhibition of microglia activation, for example, as recently shown *in vitro* and *in vivo* using minocycline, may protect the brain after ischemic stroke by improving BBB viability and integrity [64]. It is possible that glial cell activation in ALS could lead to vessel leakage. Additionally, decreased numbers of delineated astrocytes and their perivascular end-feet at the blood capillaries could affect vessel permeability.

Thus, our results confirm our previous ultrastructural findings on disruption of the BSCB showing functional incompetence of BSCB structural elements in ALS mice. A breakdown in the BSCB is clearly indicated by EB leakage in cervical/lumbar spinal cord microvessels in G93A mice at early and late stages of disease. Laminin labeling suggests that basement membrane of vessels in the spinal cords of the diseased G93A mice may be affected. Additionally, downregulation of Glut-1 and CD146 expressions in the endothelial cells of the BSCB may be related to altered endothelial lining leading to vascular leakage. Degeneration of astrocytes could influence BSCB integrity. Importantly, is BSCB breakdown a primary or secondary mechanism to motor neuron degeneration in G93A mice? Demonstrating BSCB disruption prior to the onset of disease symptoms and other pathological processes would indicate that BSCB disruption plays a primary role in ALS pathogenesis.

## Materials and Methods

### Animals

All described procedures were approved by the Institutional Animal Care and Use Committee at USF and conducted in compliance with the *Guide for the Care and Use of Laboratory Animals*. Transgenic male mice B6SJL-TgN (SOD1-G93A) 1GUR (G93A; Jackson Laboratories), over-expressing human SOD1, carrying the Gly93→Ala mutation, were used. Fourteen mice at 7 weeks of age were assessed on sensitive indicators of degenerative state (body weight and extension reflex). Six C57BL/6J male mice of the same age were used as controls. All mice were maintained on a 12∶12 h dark∶light cycle (light on at 06:00 hours). Room temperature was 23°C. Food and water were available *ad libitum.*

### Characteristics of disease progression

Body weight was measured weekly throughout the study. Extension reflex was also observed weekly, as we previously described [46]. Briefly, extension of the hindlimbs was observed while the mouse was suspended by its tail. A score (0–2) was given to each mouse indicating: normal (2), partial (1) or absent (0) hindlimb extension. Eight G93A mice were euthanatized at initial signs of disease (tremor, weight loss, and reduced hindlimb extension) at approximately 13 weeks of age, while six were allowed to reach the end-point of hindlimb paralysis at 17–18 weeks of age, when they were also euthanatized. Measures of body weight and extension reflex were recorded for C57BL/6J mice according to the same schedule as G93A mice. C57BL/6J mice were euthanatized at 12–13 weeks (n = 3) or 19–20 weeks (n = 3) of age.

### Evans Blue dye

Evans Blue dye (EB, Aldrich Chemical), 961 Da, was used as a tracer for assessing BBB disruption [65]. G93A mice with initial or late stage disease and C57BL/6J mice were intravenously injected with 2% EB in saline solution via the jugular vein 30–40 min prior to euthanasia. This surgical procedure was performed as we previously described [46]. Briefly, mice were anesthetized with Isoflurane delivered using a calibrated vaporizer equipped induction chamber and nose cone and administered at 2–5% in O_2_ (2 L/min) to induce anesthesia and then decreased to 2% to maintain the anesthesia. The jugular vein was exposed and isolated using blunt dissection. The vein was ligated and a 31-gauge needle, attached to a 100-µl Hamilton syringe, was placed into the lumen of the vein and sutured in place. Evans Blue was delivered (0.2 ml/100g, 40 µl/mouse) during 2 min. The needle was withdrawn, the suture tightened, and the incision closed with Vetbond.

### Euthanatasia and tissue preparation

Euthanatasia of all mice was achieved under deep pentobarbital anesthesia and perfusion was not performed to avoid mechanical disruption of blood capillaries. The cervical/lumbar spinal cords were removed, fixed in 4% paraformaldehyde (PFA) in 0.1 M phosphate buffer (PB), pH 7.2, and then cryoprotected in 20% sucrose in 0.1 M PB (pH 7.2) overnight. Coronal sections of the cervical (C2–C3) and lumbar (L4–L5) spinal cords were cut at 30 µm in a cryostat.

### Immunofluorescence staining

For identification of EB leakage, serial tissue sections of the spinal cords were thaw-mounted on slides, washed with deionized water to remove the freezing medium, and then rinsed several times in phosphate-buffered saline (PBS). The slides were coverslipped with Vectashield (Vector) and examined under an epifluorescence microscope.

Some spinal cord tissues were used for immunofluorescent analysis of the basement membrane (laminin), endothelial cells (Glut-1, CD146), and astrocytes (GFAP). Briefly, sections of the cervical/lumbar spinal cords were labeled with rat anti-mouse laminin-1 (α and β chains) monoclonal antibody (1∶50, Chemicon), rabbit anti-mouse Glut-1 polyclonal antibody (1∶100, Alpha Diagnostic Int.), or double-stained with mouse monoclonal CD146 (1∶30, Chemicon) and rabbit polyclonal antibodies against glial fibrillary acidic protein (GFAP, 1∶500, Dako). The next day, the slides were incubated for 2 hrs with appropriate secondary antibodies conjugated to either rhodamine (1∶1500, Alexa 594, Molecular Probes) or FITC (1∶500–700, Alexa 488, Molecular Probes) and, after several rinses in PBS, coverslipped with Vectashield or Vectashield with DAPI (Vector) and examined under epifluorescence using an Olympus BX60 microscope.

### Staining of motor neurons in the spinal cord

Coronal sections of the C2–C3 and L4–L5 spinal cords were rinsed in PBS and then stained with 0.1% cresyl violet (30 sec) for routine histological analysis of motor neurons. Sections were then washed several times in deionized water, air-dried, dehydrated, and coverslipped using Permount.

### Statistical analysis

Data are presented as means±SEM. The nonparametric Mann-Whitney unpaired test was used to compare medians.
